# Rapid decrease in titer and breadth of neutralizing anti-HCV antibodies in HIV/HCV-coinfected patients who achieved SVR

**DOI:** 10.1038/s41598-019-48592-5

**Published:** 2019-08-21

**Authors:** Lorena Vigón, Sonia Vázquez-Morón, Juan Berenguer, Juan González-García, Ma Ángeles Jiménez-Sousa, Josep M. Guardiola, Manuel Crespo, Ignacio de Los Santos, Miguel A. Von Wichmann, Ana Carrero, María Belén Yélamos, Julián Gómez, Salvador Resino, Isidoro Martínez, The GESIDA 3603b Cohort Study Group, P. Miralles, J. C. López, F. Parras, B. Padilla, T. Aldamiz-Echevarría, F. Tejerina, C. Díez, L. Pérez-Latorre, C. Fanciulli, I. Gutiérrez, M. Ramírez, S. Carretero, J. M. Bellón, J. Bermejo, V. Hontañón, J. R. Arribas, M. L. Montes, I. Bernardino, J. F. Pascual, F. Zamora, J. M. Peña, F. Arnalich, M. Díaz, P. Domingo, J. Sanz, M. J. Bustinduy, J. A. Iribarren, F. Rodríguez-Arrondo, E Van den Eynde, M. Pérez, E. Ribera, J. L. Casado, F. Dronda, A. Moreno, M. J. Pérez-Elías, M. A. Sanfrutos, S. Moreno, C. Quereda, A. Arranz, E. Casas, J. de Miguel, S. Schroeder, J. Vergas, M. J. Téllez, D. Vinuesa, L. Muñoz, J. Hernández-Quero, A. Ferrer, M. J. Galindo, L. Ortiz, E. Ortega, M. Montero, M. Blanes, S. Cuellar, J. Lacruz, M. Salavert, J. López-Aldeguer, G. Pérez, G. Gaspar, M. Yllescas, P. Crespo, E. Aznar, H. Esteban

**Affiliations:** 1 Unidad de Infección Viral e Inmunidad, Centro Nacional de Microbiología, Instituto de Salud Carlos III, Majadahonda, Madrid Spain; 2 Unidad de Enfermedades Infecciosas/VIH; Hospital General Universitario “Gregorio Marañón”, Madrid, Spain; 3 Instituto de Investigación Sanitaria del Gregorio Marañón, Madrid, Spain; 4 Unidad de VIH, Servicio de Medicina Interna, Hospital Universitario “La Paz”, Madrid, Spain; 5 Hospital Santa Creu i Sant Pau, Barcelona, Spain; 6 Hospital Alvaro Cunqueiro, Vigo, Pontevedra Spain; 7 Hospital Universitario de La Princesa, Madrid, Spain; 8 Hospital Universitario Donostia, San Sebastián, Gipuzkoa Spain; 9 Departamento de Bioquímica y Biología Molecular, Facultad de Ciencias Químicas, Universidad Complutense, Madrid, Spain; 10 Hospital Universitari Vall d’Hebron, Barcelona, Spain; 11 Hospital Universitario Ramón y Cajal, Madrid, Spain; 12 Hospital Universitario Príncipe de Asturias, Alcalá de Henares, Spain; 13 Hospital Clínico San Carlos, Madrid, Spain; 14 Hospital Universitario San Cecilio, Granada, Spain; 15 Hospital Clínico Universitario, Valencia, Spain; 16 Hospital General Universitario, Valencia, Spain; 17 Hospital Universitari La Fe, Valencia, Spain; 18 Hospital Universitario de Getafe, Getafe, Spain; 19 Fundación SEIMC-GESIDA, Madrid, Spain

**Keywords:** Hepatitis C, Antibodies, Hepatitis C virus

## Abstract

The main targets for neutralizing anti-hepatitis C virus (HCV) antibodies (HCV-nAbs) are the E1 and E2 envelope glycoproteins. We have studied the characteristics of HCV-nAbs through a retrospective study involving 29 HIV/HCV-coinfected patients who achieved sustained virological response (SVR) with peg-IFNα + ribavirin anti-HCV therapy. Plasma samples at baseline and week 24 after SVR were used to perform neutralization assays against five JFH1-based HCV recombinant viruses coding for E1 and E2 from genotypes 1a (H77), 1b (J4), 2a (JFH1), 3a (S52) and 4a (ED43). At baseline, the majority of plasma samples neutralized 1a, 1b, 2a, and 4a, but not 3a, genotypes. Twenty-four weeks following SVR, most neutralizing titers declined substantially. Furthermore, titers against 3a and 2a were not detected in many patients. Plasma samples with high HCV-nAb titers neutralized all genotypes, and the highest titers at the starting point correlated with the highest titers at week 24 after SVR. In conclusion, high titers of broad-spectrum HCV-nAbs were detected in HIV/HCV-coinfected individuals, however, those titers declined soon after SVR.

## Introduction

Hepatitis C virus (HCV) infection is a serious global health problem. According to a recent WHO report, 71 million people were living with chronic HCV infection worldwide in 2015^1^. These individuals are at risk of developing cirrhosis, end-stage liver disease, and hepatocellular carcinoma over time^2,3^. Despite the impressive efficacy of the direct-acting antivirals (DAAs) against HCV infection (>90% achieved sustained virological response [SVR]), there are still some problems facing the hope of controlling HCV infection with DAAs alone^4^. Since hepatitis C is largely asymptomatic in the initial stages, less than 20% of HCV-infected individuals worldwide are aware that they are infected and only a minority of them have access to anti-HCV treatment and thus, remain at risk of transmitting the HCV infection^1^. Furthermore, those that receive successful DAAs therapy are not protected against reinfection with HCV^5^. For these reasons, the development of an effective preventive vaccine will provide a useful, cost-effective tool at attempts to control and eradicate HCV infection^6^.

HCV is a positive-strand RNA virus with a nucleocapsid containing the HCV genome and an envelope crowned by the E1and E2 envelope glycoproteins, which are the main targets for neutralizing anti-HCV antibodies (HCV-nAbs)^7^. Several lines of evidence point to the involvement of HCV-nAbs in protecting from HCV infection: a rapid and potent HCV-nAbs response correlates with spontaneous HCV clearance^8–12^, and the passive administration of anti-HCV antibodies prevents HCV infection in experimental animals^13–17^. Therefore, studies monitoring titers and dynamics of HCV-nAbs are pertinent for designing strategies aimed to protect people from HCV infection, particularly in at-risk populations (injecting drug users [IDU] and men who have sex with men [MSM], among others).

Both primary HCV infection and HCV reinfections are common among human immunodeficiency virus (HIV)-infected individuals within the IDU and MSM groups^18,19^. However, little is known about the dynamics of HCV-nAbs in HIV-infected patients who eliminate HCV infection after HCV therapy.

In this study, we have analyzed the titers, breadth, and dynamic of HCV-nAbs in HIV/HCV-coinfected individuals before HCV treatment and after achieving SVR with HCV therapy.

## Results

### Patient characteristics at baseline

Table 1 shows the characteristics of each of the 29 HIV/HCV-coinfected patients at baseline. Overall, the median age was 49 years, 69% were male, 75.9% were IDU, and 27.6% had prior acquired immune deficiency syndrome (AIDS)-defining conditions. All patients were on combined antiretroviral therapy (cART), and all had undetectable HIV viral load (<50 copies/ml). The median count of CD4+ T-cells was 695 cells/mm3 and only eight patients had CD4+ T-cells < 500 cells/mm3. Regarding chronic hepatitis C, two patients were F0-F1 (<7.1 Kpa), five were F2 (7.1–9.5 Kpa), eight were F3 (9.5–12.5 Kpa), and 14 were F4 (≥12.5 Kpa). The median of log_10_ HCV-RNA was 6.62 IU/ml; 19 patients had log_10_ HCV-RNA values ≥ 6 IU/ml; 14 had had previous HCV therapy (median of 92 months before the baseline); and 13 patients were coinfected with HCV-GT1a, three with HCV-GT1b, eight with HCV-GT3 and five with HCV mixed infection.

**Table 1:** Epidemiological and clinical characteristics at baseline of the HIV/HCV-coinfected patients included in the study.

#	Age (years)	Gender	Previous HCV therapy	Time to baseline (months)	Nadir CD4^+^ T (cells/mm^3^)	Prior AIDS	IDU	CD4^+^/CD8^+^	CD4^+^ T cells/mm^3^	HIV-RNA < 50 cp/mL	HCV genotype	Log_10_ HCV-RNA (IU/mL)	LSM (kPa)
29	45	Male	—	—	214	Yes	No	1.26	814	Yes	1a/2	6.39	6.7
37	55	Male	Peg-IFNα/RBV	106	41	Yes	Yes	2.32	518	Yes	1a	7.10	9.8
38	42	Male	Peg-IFNα/RBV	14	24	Yes	Yes	1.00	1053	Yes	1a/4	5.73	17.8
48	49	Female	—	—	174	Yes	No	1.69	916	Yes	3	6.13	35.8
49	53	Male	—	—	60	Yes	Yes	0.83	523	Yes	1a	5.87	10.8
95	48	Female	—	—	60	No	Yes	0.54	280	Yes	1a/1b	6.04	20.6
96	53	Male	—	—	260	No	Yes	0.93	530	Yes	3	7.18	9.5
97	52	Male	Peg-IFNα/RBV	120	675	No	Yes	1.09	810	Yes	1a	6.76	11.9
103	54	Male	Peg-IFNα/RBV	28	90	No	No	0.98	348	Yes	1b	4.54	21.3
109	46	Female	Peg-IFNα/RBV	99	150	No	Yes	1.35	896	Yes	1a	6.69	24.8
114	36	Male	—	—	314	No	No	0.87	887	Yes	1a	6.49	8.8
115	52	Male	—	—	277	No	No	0.99	706	Yes	3	7.17	10.1
133	45	Male	—	—	421	No	Yes	1.38	695	Yes	3	7.00	4.9
139	57	Male	—	—	48	Yes	Yes	0.70	447	Yes	1a	4.36	7.9
148	53	Male	IFNα/RBV	86	234	No	Yes	0.81	372	Yes	1a	5.83	7.8
174	40	Female	—	—	122	No	Yes	0.84	662	Yes	4/1a	6.46	13.8
213	50	Male	—	—	323	No	Yes	0.79	1210	Yes	3	6.47	25.4
216	50	Female	—	—	117	No	Yes	2.12	1008	Yes	3/4	6.23	10.4
432	49	Female	—	—	154	Yes	Yes	1.40	925	Yes	3	5.74	7.6
515	47	Male	Peg-IFNα/RBV	23	84	No	Yes	0.44	390	Yes	3	6.38	39.1
711	47	Male	—	—	46	No	Yes	2.09	200	Yes	1a	6.00	12.8
718	44	Female	Peg-IFNα/RBV	47	196	No	No	0.87	578	Yes	3	6.11	13.4
719	46	Male	—	—	383	No	Yes	0.96	631	Yes	1a	4.76	11.4
721	53	Female	IFNα/RBV	17	21	No	Yes	1.00	210	Yes	1a	6.62	11.8
722	51	Male	Peg-IFNα/RBV	22	162	No	No	0.96	724	Yes	1b	6.67	18.0
724	53	Male	IFNα/RBV	93	214	No	Yes	1.96	446	Yes	1a	5.54	27.7
725	47	Male	Peg-IFNα/RBV	101	225	No	Yes	1.28	1274	Yes	1a	6.46	14.5
726	43	Female	Peg-IFNα/RBV	67	303	No	Yes	1.26	762	Yes	1a	5.67	10.2
729	47	Male	IFNα/RBV	68	261	Yes	Yes	1.33	1218	Yes	1b	6.46	13.2

Abbreviations: AIDS, acquired immune deficiency syndrome; IDU, injecting drug user; HCV, hepatitis C virus; HCV-RNA, HCV plasma viral load; HIV, human immunodeficiency virus; HIV-RNA, HIV plasma viral load; LSM, liver stiffness measure; kPa, kilopascals; IFNα, interferon-alpha; peg-IFNα, PEGylated interferon-alpha; RBV, ribavirin.

Table 2 shows the characteristics of antiviral therapy in HIV/HCV-coinfected patients during the follow-up. All patients were treated with peg-IFNα + ribavirin (RBV), and DAAs were administered to 11 patients (Telaprevir to seven patients and Sofosbuvir to four patients). The majority of patients received anti-HCV treatment for 48 weeks. In addition, all patients were treated against HIV infection. At the end of follow-up, the median of CD4+ T-cells was 827 cells/mm^3^, and only seven patients had HIV viral load >50 copies/ml.

**Table 2:** Characteristics of the antiviral therapy in HIV/HCV-coinfected patients included in the study during the follow-up.

	HCV therapy			Antiretroviral therapy				HIV biomarkers at the end of study	
Patient #	IFNα therapy	DAAs	Time on HCV therapy (wks.)	cART	Drug #1	Drug #2	Drug #3	CD4^+^ T cells/mm^3^	HIV-RNA (copies/mL)
29	Peg-IFNα/RBV	—	48	2PI + II	Atazanavir/r	Raltegravir		1177	224
37	Peg-IFNα/RBV	—	48	2NRTI + NNRTI	Abacavir	Lamivudine	Etravirine	827	<50
38	Peg-IFNα/RBV	Telaprevir	48	2PI + II	Atazanavir/r	Raltegravir		955	<50
48	Peg-IFNα/RBV	—	48	NNRTI + NRTI + II	Tenofovir	Raltegravir	Etravirine	1398	<50
49	Peg-IFNα/RBV	Telaprevir	24	2NRTI + II	Tenofovir	Emtricitabine	Raltegravir	609	<50
95	Peg-IFNα/RBV	Telaprevir	48	2NRTI + NNRTI	Tenofovir	Emtricitabine	Etravirine	260	78
96	Peg-IFNα/RBV	—	48	2NRTI + 2PI	Tenofovir	Emtricitabine	Atazanavir/r	830	367
97	Peg-IFNα/RBV	Telaprevir	48	2NRTI + II	Tenofovir	Emtricitabine	Raltegravir	580	523
103	Peg-IFNα/RBV	Telaprevir	48	2NRTI + NNRTI	Emtricitabine	Tenofovir	Etravirine	304	<50
109	Peg-IFNα/RBV	Telaprevir	48	2NRTI + NNRTI	Lamivudine	Abacavir	Etravirine	1039	<50
114	Peg-IFNα/RBV	—	48	2NRTI + NNRTI	Tenofovir	Emtricitabine	Rilpivirine	1163	<50
115	Peg-IFNα/RBV	—	48	2NRTI + NNRTI	Tenofovir	Emtricitabine	Efavirenz	637	<50
133	Peg-IFNα/RBV	—	48	2NRTI + PI	Tenofovir	Emtricitabine	Atazanavir/r	597	<50
139	Peg-IFNα/RBV	Telaprevir	48	2NRTI + II	Raltegravir	Lamivudine	Abacavir	603	<50
148	Peg-IFNα/RBV	—	36	2NRTI + II	Tenofovir	Emtricitabine	Raltegravir	726	<50
174	Peg-IFNα/RBV	—	48	PI	Darunavir/r			840	<50
213	Peg-IFNα/RBV	—	48	2NRTI + PI	Lamivudine	Abacavir	Atazanavir/r	1118	<50
216	Peg-IFNα/RBV	—	48	2NRTI + NNRTI	Emtricitabine	Efavirenz	Tenofovir	1144	<50
432	Peg-IFNα/RBV	Sofosbuvir	24	PI	Darunavir/r			1150	<50
515	Peg-IFNα/RBV	Sofosbuvir	24	2NRTI + II	Tenofovir	Emtricitabine	Raltegravir	488	<50
711	Peg-IFNα/RBV	—	48	2NRTI + II	Abacavir	Lamivudine	Raltegravir	120	<50
718	Peg-IFNα/RBV	Sofosbuvir	24	PI	Darunavir/r			790	<50
719	Peg-IFNα/RBV	Sofosbuvir	24	PI	Darunavir/r			870	<50
721	Peg-IFNα/RBV	—	36	2NRTI + II	Lamivudine	Abacavir	Raltegravir	210	99000
722	Peg-IFNα/RBV	—	48	2NRTI + II	Tenofovir	Emtricitabine	Raltegravir	660	190
724	Peg-IFNα/RBV	—	48	2NRTI + II	Raltegravir	Emtricitabine	Tenofovir	350	<50
725	Peg-IFNα/RBV	—	48	2NRTI + II	Tenofovir	Emtricitabine	Raltegravir	1330	<50
726	Peg-IFNα/RBV	—	48	2NRTI + II	Lamivudine	Abacavir	Raltegravir	870	<50
729	Peg-IFNα/RBV	—	48	2NRTI + II	Tenofovir	Emtricitabine	Raltegravir	1150	<50

Abbreviations: HCV, hepatitis C virus; HIV, human immunodeficiency virus; HIV-RNA, HIV plasma viral load; peg-IFNα, PEGylated interferon-alpha; RBV, ribavirin; DAAs, direct-acting antivirals; cART, combined antiretroviral therapy; NRTI, nucleoside analogue HIV reverse; NNRTI, non-nucleoside analogue HIV reverse transcriptase inhibitor; PI, protease inhibitor; II, integrase inhibitor.

### Potency and breadth of neutralizing antibodies against HCV chimeric viruses

Plasma samples from 29 HIV/HCV-coinfected patients were screened for neutralization potency and breadth against a panel of five JFH1-based chimeric HCVs expressing E1and E2 glycoproteins from 1a (H77), 1b (J4), 2a (JFH1), 3a (S52) and 4a (ED43) genotypes (see Supplementary Fig. 1). Most plasma samples were able to neutralize 1a (H77), 1b (J4), 2a (JFH1), and 4a (ED43) viruses with variable potency, both at baseline and 24 weeks after SVR, while S52 (3a) was only minimally neutralized, in accordance with previous reports^20^. Furthermore, patients infected with a particular HCV genotype did not show higher neutralization titers against the chimeric virus containing glycoproteins from the same genotype compared to the other chimeric viruses carrying glycoproteins from a different genotype (see Supplementary Fig. 1). In other words, similar patterns of neutralization were observed in most of the patients, regardless of the infecting genotype. To confirm that the genetic distance between the viruses did not correlate with the neutralization pattern of the corresponding plasma samples, the E1E2 genes from eight patient-derived 1a viruses were amplified and sequenced. All eight sequences clustered with H77 (1a) and other 1a viruses (Supplementary Fig. 2). However, the corresponding plasma samples had higher HCV-nAb titers against J4 (1b) and ED43 (4a) than against H77 (1a) (Supplementary Fig. 1), confirming that there was no correlation between the genetic distance between the infecting viruses and the neutralizing capacity of the antibodies from the infected individuals.

**Figure 1:**
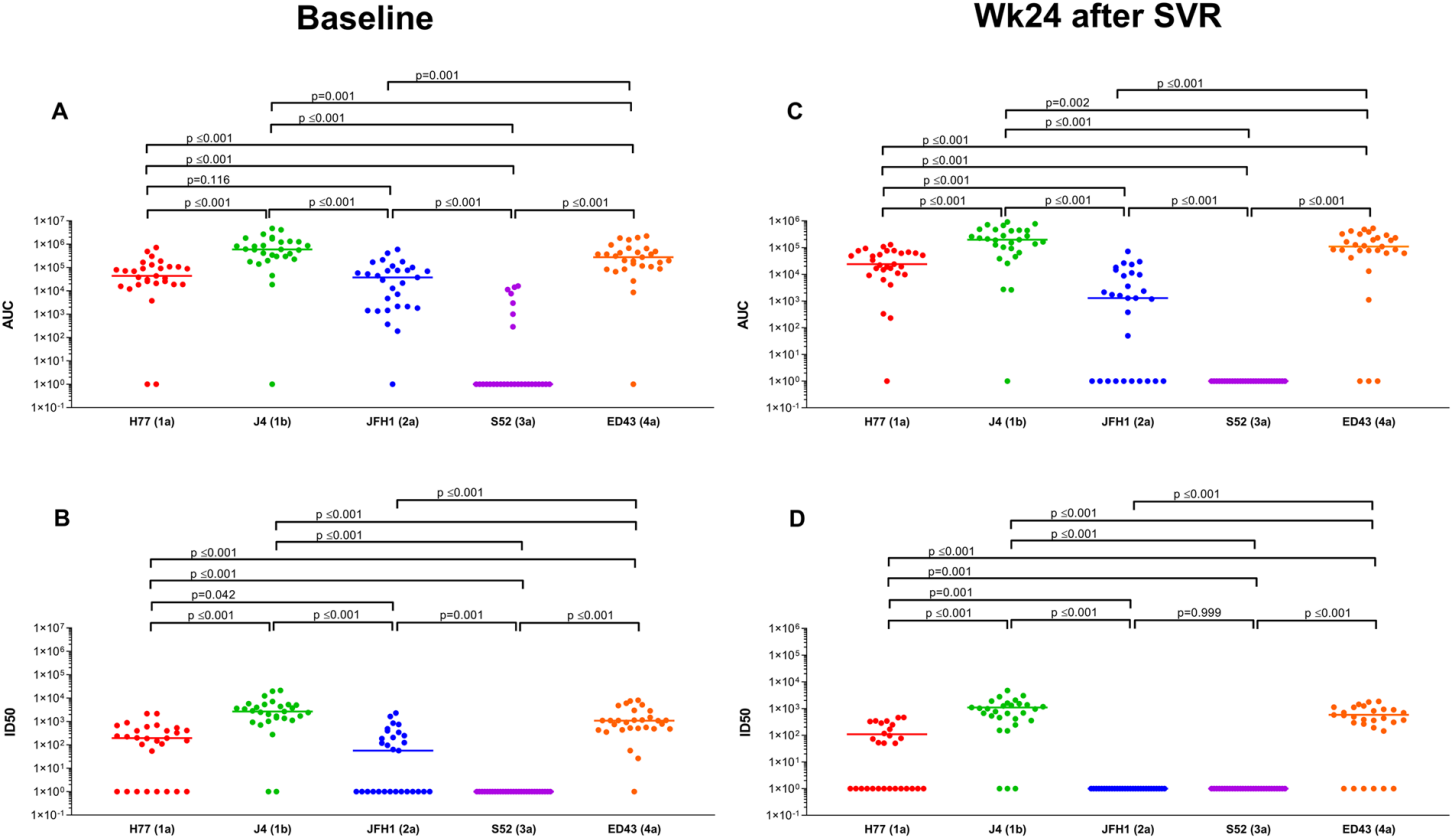
Summary of the neutralization potency of HCV antibodies against recombinant viruses in HIV/HCV-coinfected patients, at baseline and 24 weeks after sustained virological response. Individual (circles) and median values (short horizontal bars) are represented. Abbreviations: AUC, area under the curve (arbitrary units); ID50, 50% inhibitory dose; SVR, sustained virological response; Wk, week. Statistics: p-values were calculated by the Wilcoxon test.

The neutralization potency against recombinant viruses was quantified by the AUC (see Supplementary Table 1) and the ID50 (see Supplementary Table 2). At baseline, median values for both AUC (Fig. 1A) and ID50 (Fig. 1B) had the following decreasing order of potency: J4 (1b) > ED43 (4a) > H77 (1a) > JFH1 (2a) > S52 (3a). At week 24 after SVR, the same decreasing order for the AUC (Fig. 1C) and the ID50 (Fig. 1D) was observed. However, it is important to note that HCV-nAbs against S52 (3a) were not detected in any of the patients at week 24 after SVR (Fig. 1C,D). Similarly, HCV-nAbs against JFH1 (2a) were also not detected in many patient samples from week 24 after SVR (Fig. 1C,D).

**Figure 2:**
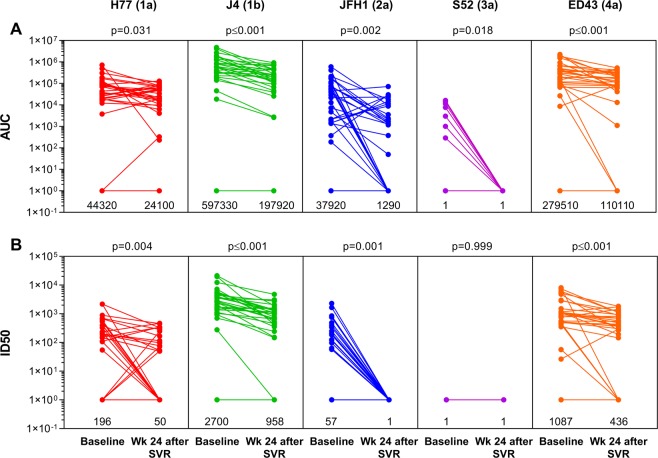
Impact of HCV clearance on HCV-nAb titers in HIV/HCV-coinfected patients who achieved sustained virological response. Abbreviations: AUC, area under the curve (arbitrary units); ID50, 50% inhibitory dose; SVR, sustained virological response; Wk, week. Statistics: p-values were calculated by the Wilcoxon test. Median values of AUC and ID50 are shown under each aligned dot plot.

The impact of HCV clearance on the HCV-nAb titers was evaluated through an analysis of repeated measures (Fig. 2). We found significant decreases in AUC (p ≤ 0.05; Fig. 2A) and ID50 (p ≤ 0.05; Fig. 2B) values for all HCV recombinant viruses at week 24 after SVR, except for ID50 values from the S52 (3a) virus because all values were null.

To confirm that anti-HCV antibodies declined shortly after anti-HCV treatment, the reactivity of the 17 plasma samples positive for HCV-GT1a was tested at baseline and week 24 after SVR against the purified E2 glycoprotein from H77 (HCV-GT1a) in an enzyme-linked immunosorbent assay. Like HCV-nAbs, total antibodies against E2 were substantially reduced at week 24 after SVR **(**Supplementary Fig. 3**)**.

### Correlation analysis among HCV-nAb titers against chimeric viruses

**Figure 3:**
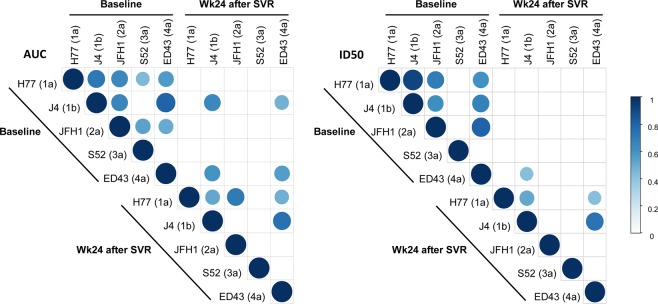
Summary of relevant correlation coefficients (r > 0.4; p < 0.05) among HCV-nAb titers against recombinant viruses in HIV/HCV-coinfected patients at baseline and 24 weeks after sustained virological response. The correlation coefficients are denoted by the intensity of the color and the size of the circle. Abbreviations: AUC, area under the curve (arbitrary units); ID50, 50% inhibitory dose; SVR, sustained virological response; Wk, week. Statistics: Correlation coefficients and p-values were calculated by the test of Spearman’s correlation coefficient.

At baseline, the AUC values for a particular chimeric virus correlated positively with the corresponding values from the other chimeric viruses (Fig. 3). That is, patients with high HCV-nAb titers against a specific chimeric virus also had high titers against the other chimeric viruses (r > 0.4; p ≤ 0.05). The lowest correlations were found between S52 (3a) and J4 (1b), and between S52 (3a) and ED43 (4a) (Fig. 3). Similarly, a positive correlation was also observed among HCV-nAb titers (ID50) against H77 (1a), J4 (1B), JFH1 (2a), and ED43 (4a) (r > 0.4; p ≤ 0.05); while S52 (3a) did not show any correlation with the other chimeric viruses (Fig. 3).

At week 24 after SVR, HCV-nAb titers (AUC) against J4 (1b) and ED43 (4a) correlated positively with baseline values for J4 (1b) and ED43 (4a) (r > 0.4; p ≤ 0.05), indistinctly (Fig. 3). This means that patients with the highest HCV-nAb titers at the end of follow-up were those who also had the highest HCV-nAb titers at baseline. Furthermore, at week 24 after SVR, HCV-nAb titers against H77 (1a) correlated with HCV-nAb titers against J4 (1b), JFH1 (2a), and ED43 (4a); and HCV-nAb titers against J4 (1b) also correlated with HCV-nAb titers against ED43 (4a) (Fig. 3). When we analyzed the correlations among ID50 values, a similar pattern as that observed for the AUC values was found, but the number of relevant correlations was lower (Fig. 3).

### Correlation among clinical data and HCV-nAb titers

At baseline, we found a significant positive relationship between the CD4/CD8 ratio and the HCV-nAb titers against H77 (1a) (AUC [r = 0.454**;** p = 0.013] and ID50 [r = 0.450**;** p = 0.014]). However, we did not find any correlation between the CD4/CD8 ratio and the HCV-nAb titers at the end of the study (see Supplementary Table 3). We also looked for correlations with other clinical variables (age, nadir CD4+ T cells/mm^3^, CD4+ T cells/mm^3^, log_10_ HCV-RNA, and LSM), but no significant association was found (data not shown).

## Discussion

HCV co-infection has a high impact on the morbidity and mortality among HIV infected patients as a result of a more aggressive progression of liver fibrosis and cirrhosis^21–24^. In this population, successful anti-HCV treatment with peg-IFN and ribavirin had a positive impact on these conditions^25–27^, and treatment with DAAs improved patient-reported outcomes^28–30^. However, other relevant outcomes associated with HCV elimination in HIV/HCV-coinfected patients remain to be investigated.

Since HCV-nAbs correlate with protection and virus clearance^31^, it is important to know how treatment-mediated HCV elimination may influence HCV-nAb titers after therapy. In the present study, we found several things to be true. First, most of the HIV/HCV-coinfected patients had high titers of broad HCV-nAbs at baseline, but these titers declined significantly 24 weeks after SVR, particularly for JFH1 (2a) and S52 (3a) viruses, against which HCV-nAbs were generally not detected at that time. Second, patients with high HCV-nAb titers against a particular genotype also had high titers against the other genotypes and retained moderately high HCV-nAb titers at the end of follow-up. HCV-nAb titers against S52 (3a) showed the worst correlation with HCV-nAb titers against the other genotypes. Third, the CD4/CD8 ratio correlated positively with HCV-nAb titers against H77 (1a). Overall, by measuring potency and breadth of HCV-nAbs, our study provides functionally relevant results, as these antibodies are correlated with HCV protection^13–17^ and HCV clearance^8–12^. To our knowledge, this is the first study that analyzes the evolution of HCV-nAbs in a cohort of HIV/HCV-coinfected patients who eliminated HCV infection after HCV therapy.

The high degree of genetic diversity of HCV is a major impediment to vaccine development^7^. This genetic heterogeneity is particularly relevant in the envelope glycoproteins (E1 and E2), where amino acid sequences vary up to 30% between HCV genotypes, 20% within genotypes, and 10% within subtypes^31^. Defining the neutralizing breadth of cross-reactive anti-HCV antibodies is critical to understanding the protective capacity against different HCV genotypes. In this study, most of the patients showed high levels of broad-spectrum HCV-nAbs at baseline. Overall, the HCV-nAb titers (both AUC and ID50) were as follows: J4 (1b) > ED43 (4a) > H77 (1a) > JFH1 (2a) > S52 (3a). This response pattern has been previously described in patients monoinfected by HCV, possibly due to strain-specific, rather than genotype-specific effects on the induction of HCV-nAbs^31^. Thus, the neutralizing activity of a patient’s plasma was not dictated primarily by his infecting HCV genotype, since, for example, plasma from patients infected with HCV genotype 1a or 1b neutralized HCV chimeric virus for genotype 4 quite efficiently. However, it should be noted that chimeric viruses from HCV genotypes 2a and 3a were neutralized with less efficiency, regardless of the HCV genotype of the patient. Although a correlation between the genetic distance of the infecting viruses and the neutralizing capacity of the anti-HCV antibodies from the infected patients was not found, an important point to consider is that more than one infecting genotype has been detected in five individuals. This strongly suggests that different HCV genotypes might have infected most patients along the course of their chronic infections. This probably has contributed to the development of broad-spectrum HCV-nAbs.

Another relevant finding of our work was a significant decrease in HCV-nAb titers at week 24 after SVR, suggesting that continuous exposition to HCV antigens is necessary to maintain high HCV-nAb titers. In our study, the number of patients with very low HCV-nAb titers increased at the end of follow-up, particularly for JFH1 (2a) and S52 (3a) viruses. However, although the drop in HCV-nAb titers was more pronounced in the case of H77 (1a), J4 (1b), and ED43 (4a), titers against these viruses remained relatively high at week 24 after SVR. This was because titers against these viruses were usually higher than against the other viruses at baseline. There is not much information on the dynamics of antibodies against HCV in patients who eliminated HCV infection. Wiegand *et al*. found a rapid decrease of anti-HCV antibodies in patients with acute hepatitis C who were treated with IFN-α-2b and eliminated the HCV infection^32^. However, patients chronically infected with HCV who eliminated HCV infection after IFN therapy had a slow decrease, which may be prolonged for several years^32–38^. A rapid decrease of anti-HCV antibodies was also reported in HIV/HCV-coinfected individuals following peg-IFN-α/ribavirin treatment during acute, but not chronic, HCV infection^39,40^. In contrast, our study shows a quick drop in HCV-nAbs following peg-IFN-α/ribavirin treatment of chronic HCV-infected patients coinfected with HIV. Note that an important difference between our study and previous reports is that all these preceding studies evaluated total antibodies against HCV by commercial semiquantitative immunoassays. In contrast, we evaluated HCV-nAbs or anti-E2 antibodies by quantitative methods in which serial dilutions of plasma samples were tested. Titers and breadth of HCV-nAbs might more accurately reflect the efficacy of the humoral immune response and the capacity of the patient to be protected from HCV reinfections. Thus, the decline of HCV-nAbs observed in our study after anti-HCV therapy in HIV/HCV-coinfected individuals may be one of the reasons for the very high HCV reinfection rates observed in this population, highlighting the impact of being HIV positive on the probability of HCV reinfection^41^.

HIV infection leads to the destruction and functional impairment of CD4+ T-cells, which may lead to dysfunction of the HCV-specific humoral immune response^42^. The CD4/CD8 ratio describes the overall immune dysfunction in HIV-infected patients since a low or inverted CD4/CD8 ratio is associated with altered immune function, chronic inflammation, and immune senescence^43,44^. In our study, a positive correlation between the CD4/CD8 ratio and the HCV-nAb titers was observed for H77 (1a) at baseline, but not at the end of follow-up. In agreement with this, a CD4+ T-cell-dependent reduction of HCV-nAbs has been reported in patients previously infected with HCV following incident HIV infection^45^. Additionally, Lee *et al*. found that HCV-nAbs against JFH-1 correlated positively with baseline CD4+ T-cell counts and increased after cART in HIV/HCV-coinfected patients, regardless of the baseline CD4+ T-cell counts^46^. In addition to the T-cell, HIV infection induces dysregulation of B cells and damage of lymphoid organs, which endangers the humoral immune response^47–50^. Loss of memory B cells in HIV-infected patients has been amply documented^51–53^, and it has been associated with impaired immunization responses and reduced long-term serologic immunity to measles and *Streptococcus pneumoniae*^54,55^. Importantly, antiretroviral therapy did not restore serologic memory in primary or in chronic infection^55^. These results are in line with our observation that HCV-nAbs decrease short after HCV therapy in HIV-infected patients undergoing ART, in contrast to what has been observed in patients infected only with HCV^32–38^. Immunoglobulin class switching also seems to be impaired in HIV infections^56,57^, which might result in the production of antibodies with reduced affinity maturation and limited neutralizing breadth^58,59^. Conversely, in the present study, a broadly neutralizing antibody response against HCV has been observed in HIV/HCV-coinfected patients. However, additional neutralization experiments with a larger panel of HCVs and with plasma samples from HCV-monoinfected patients are necessary to clarify this point.

### Limitations of the study

Firstly, this study has a retrospective design, data were collected retrospectively, and patients included in this study met a set of criteria for starting HCV treatment (see Patients and Methods), which may have introduced a selection bias. Secondly, this study has a limited number of patients, although the design with repeated measures considerably improves the statistical power of the study. Thirdly, a group of HCV-monoinfected patients was not used as a comparator to HIV/HCV-coinfected patients. Although it was not possible to include this group in our study, we would like to emphasize that several studies have reported a slow decrease of anti-HCV antibody titers following the anti-HCV treatment that may take several years^32–38^. However, these studies used semiquantitative assays and did not measure neutralizing antibodies, as it has been done in the present work. Fourthly, since peg-IFN-α + ribavirin therapy may have immunomodulatory effects affecting HCV-nAb titers, further studies using DAAs alone are needed. Fifthly, we did not evaluate the level of protection offered by the neutralizing antibodies measured in this study. This is an important issue, and the answer to this question will be highly relevant for the development of an antibody-based vaccine against HCV and to understand viral reinfections. Sixthly, although it has been shown that antibody titers decline after SVR, no information on memory B cell population nor residual liver immunity has been analyzed. Finally, we have used a reduced panel of recombinant HCVs for neutralization tests. The use of a larger and diverse panel of viruses could have added additional information on some aspects of the results.

## Conclusions

In conclusion, high titers of broad-spectrum HCV-nAbs were observed in HIV/HCV-coinfected individuals. These titers, however, declined soon after SVR. Our results suggest that those individuals may not be able to maintain protecting anti-HCV antibodies after elimination of HCV by treatment. This should be taken into account in the development of potential anti-HCV vaccines.

## Patients and Methods

### Study subjects

We performed a retrospective study (before-and-after design) in 29 HIV/HCV-coinfected patients from the cohort of “Grupo de Estudio del SIDA” (GESIDA 3603b study, Spain; see Appendix), which has been previously described^60^. All patients were treated with pegylated interferon-alpha plus ribavirin (peg-IFNα + ribavirin) or peg-IFN-α/ribavirin/DAAs between February 2012 and February 2014 and had an SVR assessment at 24 weeks after the end of HCV treatment.

Clinical, epidemiological, and virological characteristics of each patient were collected prospectively^60^. This study was performed according to the Declaration of Helsinki and was approved by the Research Ethics Committee of the Instituto de Salud Carlos III (CEI PI 23_2011) and participating centers. The participants gave their informed consent before enrollment.

We used plasma samples from baseline (between one week before starting the anti-HCV treatment and the same day at which the treatment was initiated) and the end of follow-up (week 24 after SVR, which corresponds to week 48 after the end of HCV treatment) for each patient.

### HCV genotyping

All samples were processed using the Abbott-Real Time-HCV genotype II (Abbott Laboratories, Illinois, USA) according to manufacturer instructions. First, viral RNA was extracted from 200µl of plasma sample using the QIAsymphony instrument, the commercial mini kit DSP Virus/Pathogen, and the protocol Cellfree 200 v.7. (Qiagen, Hilden, Germany). Internal, positive and negative controls from Abbott-RT-HCV II kit were incorporated into the extraction process. Then, multiplexed RT-PCR reactions were performed using the kit amplification reagent packs and the Abbott m2000rt instrument.

### E1 and E2 sequencing and phylogenetic analysis

The viral RNA extracted for HCV genotyping was used to amplify the E1E2 from eight 1a patient-derived viruses. A nested RT-PCR was carried out using the Qiagen OneStep RT-PCR Kit (Qiagen, Hilden, Germany) following manufacturer’s instructions with the following pairs of primers (numbering reference to GenBank accession AF011751)^61^: 5′GGACGGGGTAAACTATGCAACAGG3′ (nucleotide position 818–841), 5′GGGATGCTGCATTGAGTA3′ (nucleotide position 2599–2616), 5′CACCATGGGTTGCTCTTTTTCTATC3′ (nucleotide position 843–869) and 5′TTACGCCTCCGCTTGGGATATGAGTAACATCAT3′ (nucleotide position 2550–2582). The PCR products were sequenced by the Sanger method with the previous inner forward and reverse primers and two additional primers: 5′AGSGTAYTWYTCCATGG3′ (nucleotide position 1418–1434) and 5′CARCCRAACCARTTGCCC3′ (nucleotide position 1996–1979).

Phylogenetic analyses were performed using a total of 34 E1/E2 sequences corresponding to different HCV 1 obtained from GenBank public database (KP098533.1, AF009606, M62321, M67463, HQ850279, EF407457, D90208, M58335, EU781827, EU781828, D14853, AY051292, AY651061, KJ439768, KC248194, AM910652, KC248198, KC248199, KJ439772, KJ439773, KC248193, KC248196, KJ439778, KJ439782, KJ439775, KJ439781, KJ439774, HQ537007, AJ851228, KJ439780, KJ439779, KJ439776, KJ439777, NC 004102.1) and 8 E1/E2 sequences from HCV 1a infected patients. The evolutionary history was inferred using the Neighbor-Joining method^62^, a bootstrap test with 1000 replicates^63^ and the Maximum Composite Likelihood method^64^. Evolutionary analyses were conducted in MEGA7^65^. Moreover, the same analyses for these infected patients were performed with E1/E2 sequences from chimeric HCV (AB047639.1, EU204645.1, EU363760.1, EU363761.1, FJ230881.1) and a reference HCV 1a (isolate H77) (NC 004102.1).

### Cells and viruses

Human hepatoma Huh7.5 cells were obtained from Apath LLC (760 Parkside Ave. Brooklyn, NY 11226, USA) and Huh7.5.1 clone 2 from F Chisari (The Scripps Research Institute, La Jolla, USA). Cells were maintained in Dulbecco’s Modified Eagle Medium (DMEM) supplemented with 10% fetal bovine serum, 4 mM L-glutamine, 100 U/ml penicillin, and 100 U/ml streptomycin (DMEM10) at 37 °C in a 5% CO_2_ atmosphere. Plasmids encoding JFH1-based chimeric HCV genotypes 1a (H77/JFH1)^66^, 1b (J4/JFH1)^67^, 3a (S52/JFH1)^68^, and 4a (ED43/JFH1)^66^ were gifts from Jens Bukh (University of Copenhagen, Copenhagen, Denmark). The plasmid encoding HCV JFH1 (genotype 2a)^69^ was obtained from Apath LLC. Plasmids were linearized with Xba I, transcribed *in vitro* with the MEGAscript T7 kit (Invitrogen, Thermo Fisher, Rockford, IL, USA), and viruses were produced by electroporation of the resulting genomic RNA into Huh7.5.1 clone 2 cells.

### HCV neutralization assay

A total of 12,000 Huh7.5 cells per well were plated in flat-bottom 96-well tissue culture plates and incubated overnight at 37 °C. The following day, 150 focus-forming units of chimeric HCVs were mixed with an equal volume of 1:2 serial dilutions (starting at a 1:50 dilution) of the plasma sample or a control plasma from a healthy donor in DMEM10, incubated at 37 °C for 1 hour and then added to cells. After three days of infection, the cells were fixed with cold methanol for 10 minutes, followed by a one-hour incubation with the anti-NS5A 9E10 antibody (a gift from Charles Rice, The Rockefeller University, New York, USA). Foci were visualized after one-hour incubation with an anti-mouse IgG horseradish peroxidase-linked whole secondary antibody (Abcam, Cambridge, UK) and 3-amino-9-ethylcarbazole (Sigma, St. Louis, MO, USA) and counted under a light microscope. Percentage of neutralization at each antibody dilution was calculated as [1- (foci in the presence of plasma_test_/foci in the presence of plasma_control_)] × 100%. With these data, titration curves for each type of virus were made using GraphPad Prism 7.00 for Windows (GraphPad Software, La Jolla, California USA). The 50% inhibitory dose (ID50) was calculated as the dilution of plasma that resulted in a 50% reduction in the number of foci. The area under the curve (AUC) was also calculated to quantify neutralization potency^70^ by using GraphPad Prims 7.00 and the following parameters: Baseline Y = 0; ignore peaks that are less than 10% of the distance from minimum to maximum Y; all peaks must go above the baseline. The AUC is expressed as X units times the Y units.

### Production of HCV-E2 (H77) and quantitative enzyme-linked immunoassay

The ectodomain of E2 glycoprotein from genotype 1a (H77) was produced using the baculovirus/insect cell system according to a previously described procedure^71^ with slight modifications. Briefly, the DNA encoding the ectodomain of E2 (H77) protein, residues 384–661 (E2661) with the addition at the 5′ end of a six-histidine tag coding sequence, was inserted into the baculovirus transfer vector pAcGP67A (Pharmingen, San Diego, CA, USA) to create the recombinant plasmid pAcGP67A-E2661-H77. Insect *Spodoptera frugiperda* (Sf9) cells were cotransfected with flashBAC GOLDTM DNA (Oxford Expression Technologies, Oxford, UK) and the recombinant transfer vector pAcGP67A–E2661-H77 as indicated by the manufacturer. The protein was expressed by infecting High Five cells in Insect X-Press serum-free media with high titter virus (>108 pfu/ml) at MOI of 5–10. Medium with the secreted recombinant glycoprotein was collected approximately 120 h postinfection, dialyzed against 50 mM Tris-HCl pH 8.0, 50 mM NaCl, and loaded onto a Ni2+ -nitrilotriacetic acid-agarose column (Qiagen, Hilden, Germany). The recombinant E2661 protein was eluted with 200 mM imidazole in dialysis buffer.

Ninety-six-well microtiter plates were coated with 500 ng *Galanthus nivalis* lectin (GNA, Sigma, St. Louis, MO, USA) at 4 °C overnight, followed by blocking with 2% porcine serum in PBS. Then 40 ng of purified E2 were added to the wells for 2 h at room temperature, followed by incubation with plasma sample dilutions in blocking solution (dilution factor 2, initial dilution 1:50), anti-human IgG-Peroxidase (GE, Healthcare, Buckinghamshire, UK) and substrate (OPD, Sigma, St. Louis, MO, USA). Extensive washing with water was done after each step. Optical density was read at 492 nm.

### Statistical analysis

The statistical analysis was performed with the Statistical Package for the Social Sciences (SPSS) 23.0 software (IBM Corp., Chicago, USA). Statistical significance was defined as p < 0.05. All p-values were two-tailed. The Wilcoxon test was used to analyze the paired samples within each group. The correlation was analyzed using the test of Spearman’s correlation coefficient.

## Supplementary information

Supplementary information

## Data Availability

All relevant data are within the paper and Supporting Information files. For additional information, interested readers can contact Dr. Isidoro Martinez at imago@isciii.es.
