# Supplementary material for: Rapid decrease in titer and breadth of neutralizing anti-HCV antibodies in HIV/HCV-coinfected patients who achieved SVR
Source: Sci Rep. 2019 Aug 21;9:12163. doi: 10.1038/s41598-019-48592-5 (PMC6704069; doi:10.1038/s41598-019-48592-5)
Supplement: Supplementary file 1 — Supplementary information [file 41598_2019_48592_MOESM1_ESM.pdf]

Title: Rapid decrease in titer and breadth of neutralizing anti-HCV antibodies in HIV/HCV-coinfected patients who achieved SVR

Authors: Lorena VIGÓN [1], Sonia VÁZQUEZ-MORÓN [1], Juan BERENGUER [2,3], Juan GONZÁLEZ-GARCÍA [4], Mª Ángeles JIMÉNEZ-SOUSA [1], Josep M GUARDIOLA [5], Manuel CRESPO [6], Ignacio de Los SANTOS [7], Miguel A VON WICHMANN [8], Ana CARRERO [2,3], María Belén Yélamos [9], Julián Gómez [9], Salvador RESINO [1, &, *], Isidoro MARTÍNEZ [1, &,*] and The GESIDA 3603b Cohort Study Group.

(&), these authors contributed equally to this work. (*), corresponding author.

Authors' Affiliations: (1) Unidad de Infección Viral e Inmunidad, Centro Nacional de Microbiología, Instituto de Salud Carlos III, Majadahonda, Madrid, Spain. (2) Unidad de Enfermedades Infecciosas/VIH; Hospital General Universitario "Gregorio Marañón", Madrid, Spain. (3) Instituto de Investigación Sanitaria del Gregorio Marañón, Madrid, Spain. (4) Unidad de VIH, Servicio de Medicina Interna, Hospital Universitario "La Paz", Madrid, Spain. (5) Hospital Santa Creu i Sant Pau, Barcelona, Spain. (6) Hospital Alvaro Cunqueiro, Vigo, Pontevedra, Spain. (7) Hospital Universitario de La Princesa, Madrid, Spain. (8) Hospital Universitario Donostia, San Sebastián, Gipuzkoa, Spain. (9) Departamento de Bioquímica y Biología Molecular, Facultad de Ciencias Químicas, Universidad Complutense, Madrid, Spain.

Supplementary Figure 1. Neutralization potency and breadth of HCV antibodies against a panel of five JFH1-based chimeric HCVs expressing E1 andE2 glycoproteins from 1a (H77), 1b (J4), 2a (JFH1), 3a (S52) and 4a (ED43) genotypes from 29 HIV/HCV-coinfected patients, at baseline and 24 weeks after sustained virological response (SVR). Patient numbers and HCV genotypes (open squares) are shown. Curves represent one phase exponential decay least squares fit.

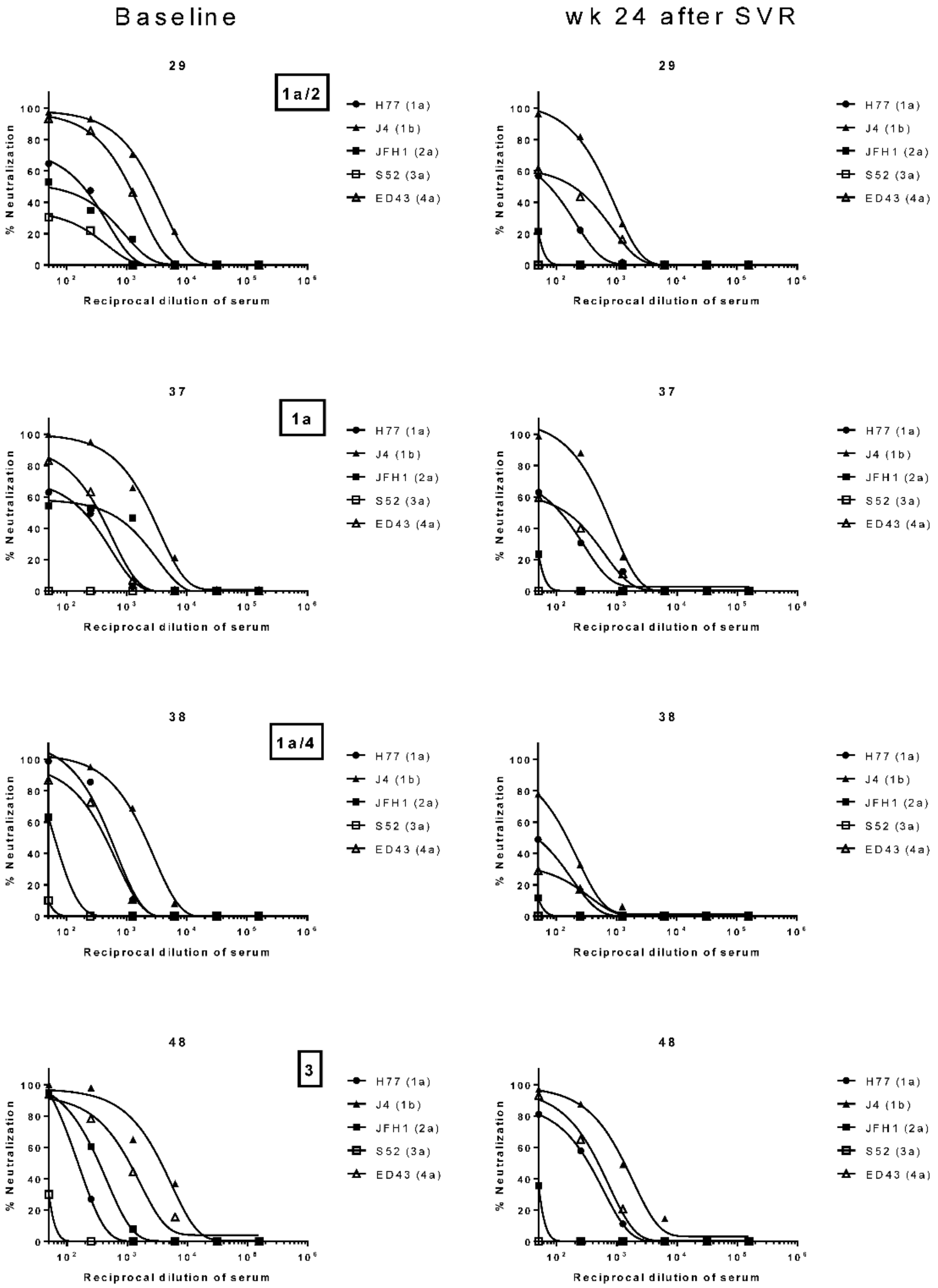

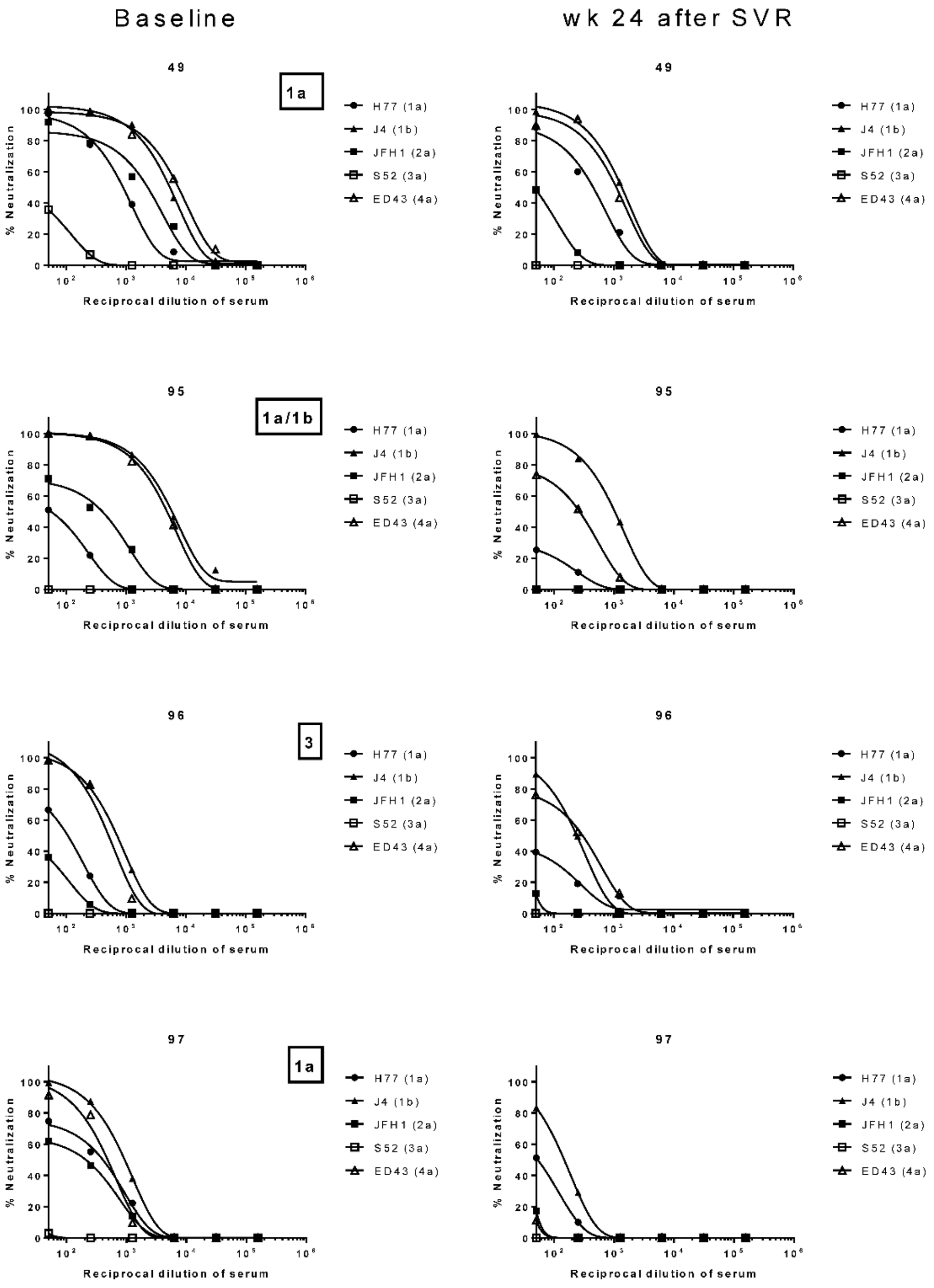

Baseline
wk 24 after SVR
49
1a
95
1a/1b
96
3
97
H77 (1a)
J4 (1b)
JFH1 (2a)
S52 (3a)
ED43 (4a)
% Neutralization
Reciprocal dilution of serum

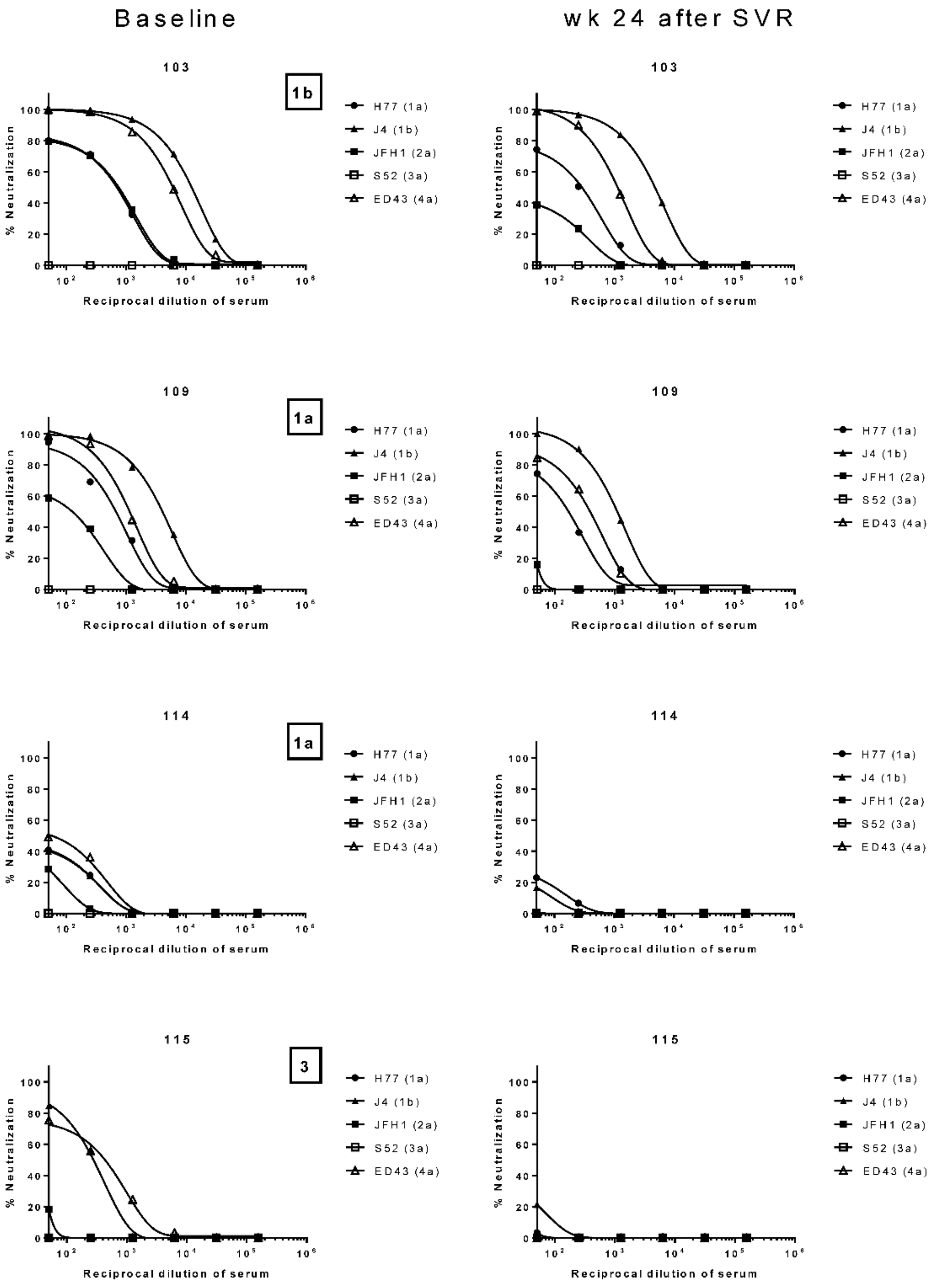
Baseline
wk 24 after SVR
103
1b
109
1a
114
1a
115
3
H77 (1a)
J4 (1b)
JFH1 (2a)
S52 (3a)
ED43 (4a)
% Neutralization
Reciprocal dilution of serum
0
20
40
60
80
100
10²
10³
10⁴
10⁵
10⁶

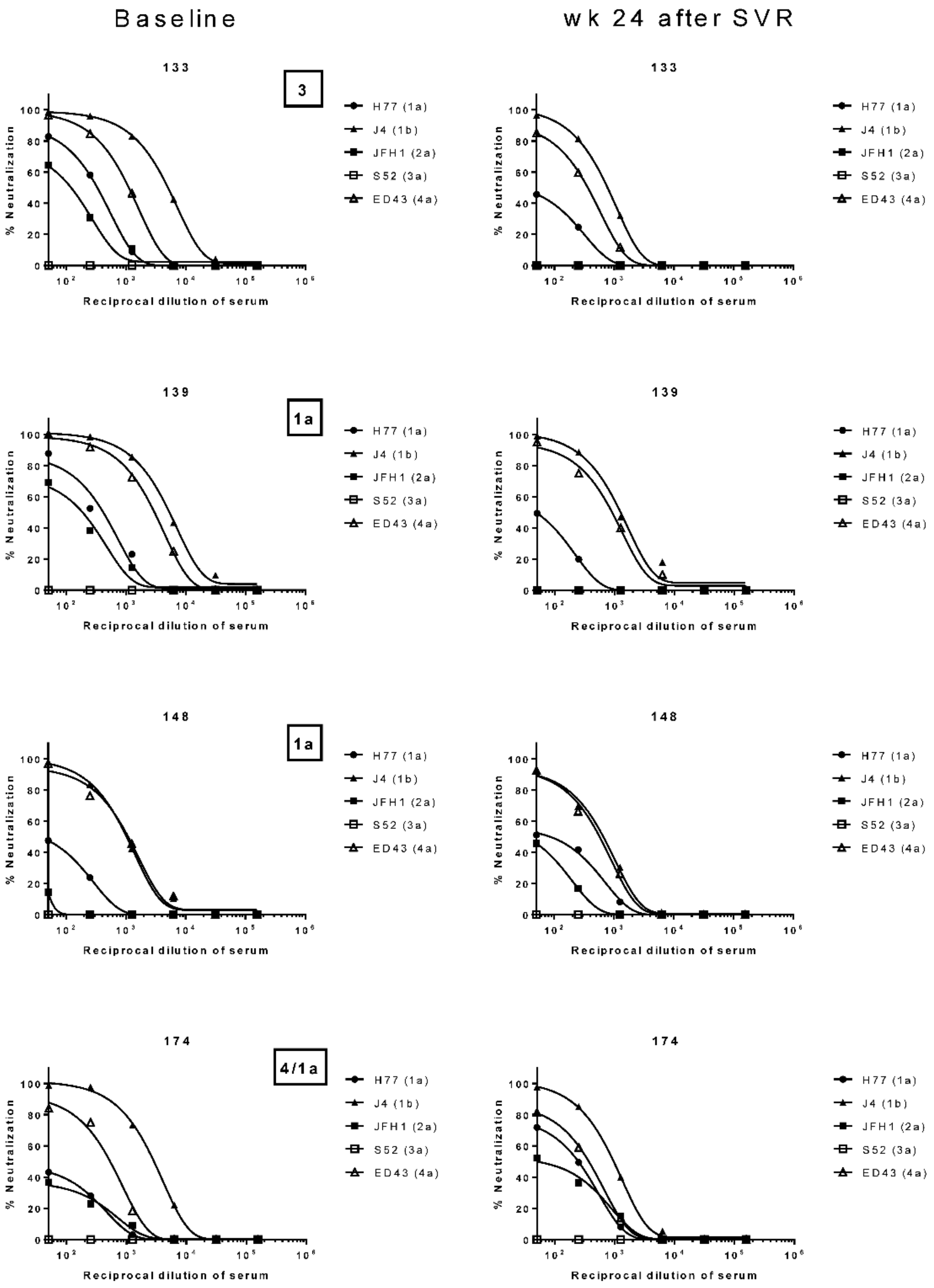
Baseline
wk 24 after SVR
133
3
139
1a
148
1a
174
4/1a
H77 (1a)
J4 (1b)
JFH1 (2a)
S52 (3a)
ED43 (4a)
% Neutralization
Reciprocal dilution of serum

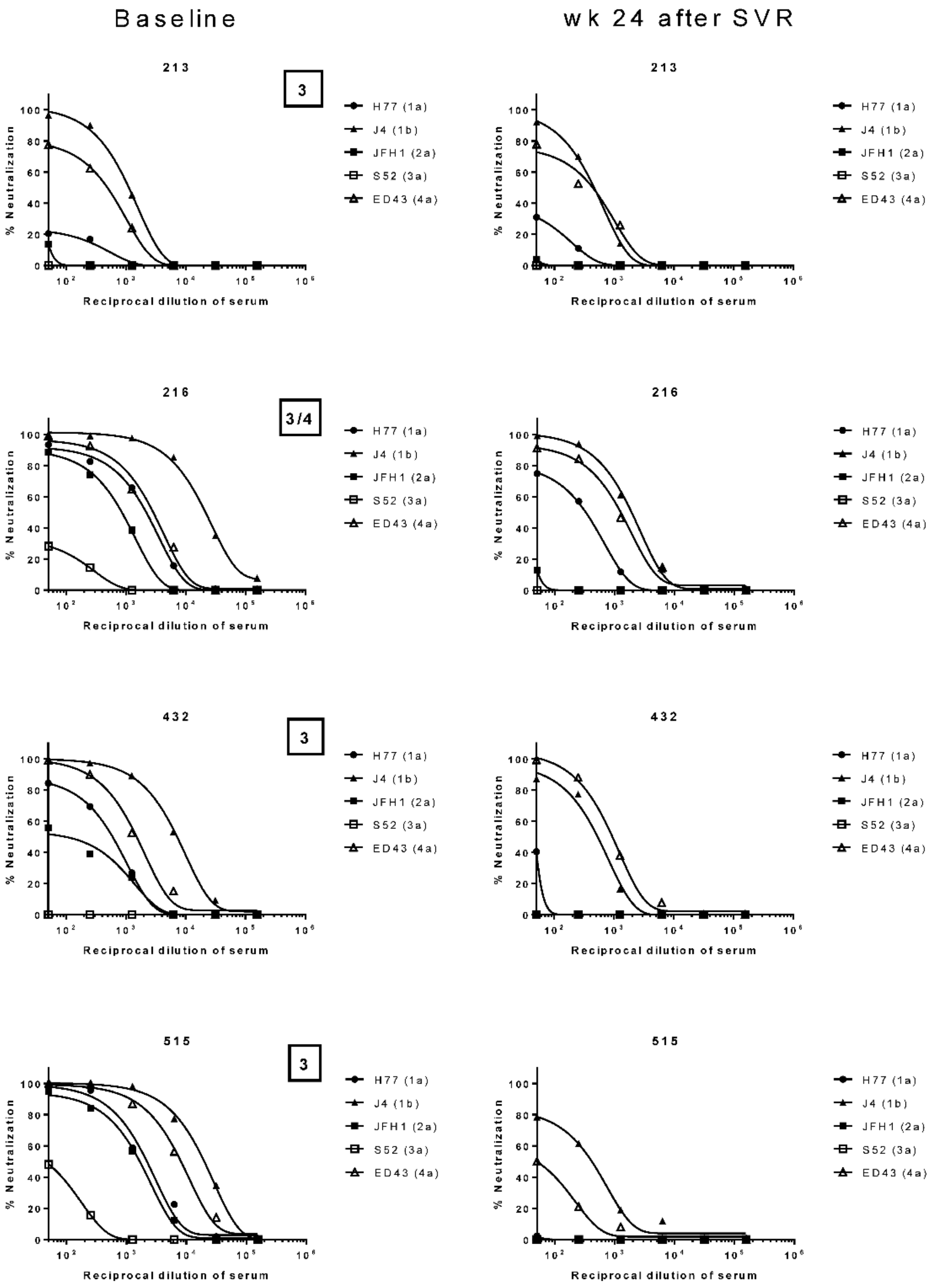
Baseline
wk 24 after SVR
213
3
216
3/4
432
3
515
3
H77 (1a)
J4 (1b)
JFH1 (2a)
S52 (3a)
ED43 (4a)
% Neutralization
Reciprocal dilution of serum

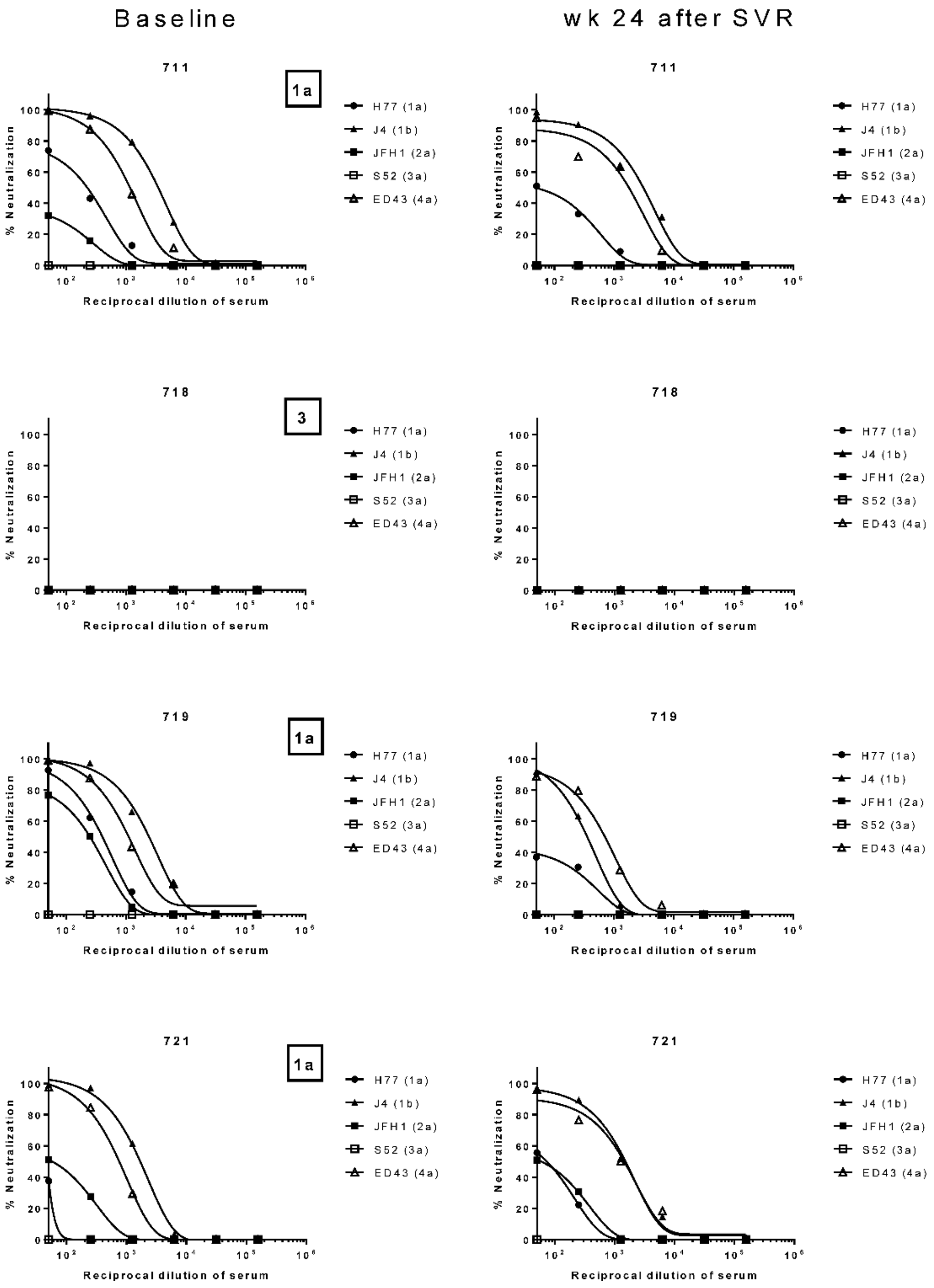
Baseline
wk 24 after SVR
711
1a
% Neutralization
Reciprocal dilution of serum
H77 (1a)
J4 (1b)
JFH1 (2a)
S52 (3a)
ED43 (4a)
711
% Neutralization
Reciprocal dilution of serum
H77 (1a)
J4 (1b)
JFH1 (2a)
S52 (3a)
ED43 (4a)
718
3
% Neutralization
Reciprocal dilution of serum
H77 (1a)
J4 (1b)
JFH1 (2a)
S52 (3a)
ED43 (4a)
718
% Neutralization
Reciprocal dilution of serum
H77 (1a)
J4 (1b)
JFH1 (2a)
S52 (3a)
ED43 (4a)
719
1a
% Neutralization
Reciprocal dilution of serum
H77 (1a)
J4 (1b)
JFH1 (2a)
S52 (3a)
ED43 (4a)
719
% Neutralization
Reciprocal dilution of serum
H77 (1a)
J4 (1b)
JFH1 (2a)
S52 (3a)
ED43 (4a)
721
1a
% Neutralization
Reciprocal dilution of serum
H77 (1a)
J4 (1b)
JFH1 (2a)
S52 (3a)
ED43 (4a)
721
% Neutralization
Reciprocal dilution of serum
H77 (1a)
J4 (1b)
JFH1 (2a)
S52 (3a)
ED43 (4a)

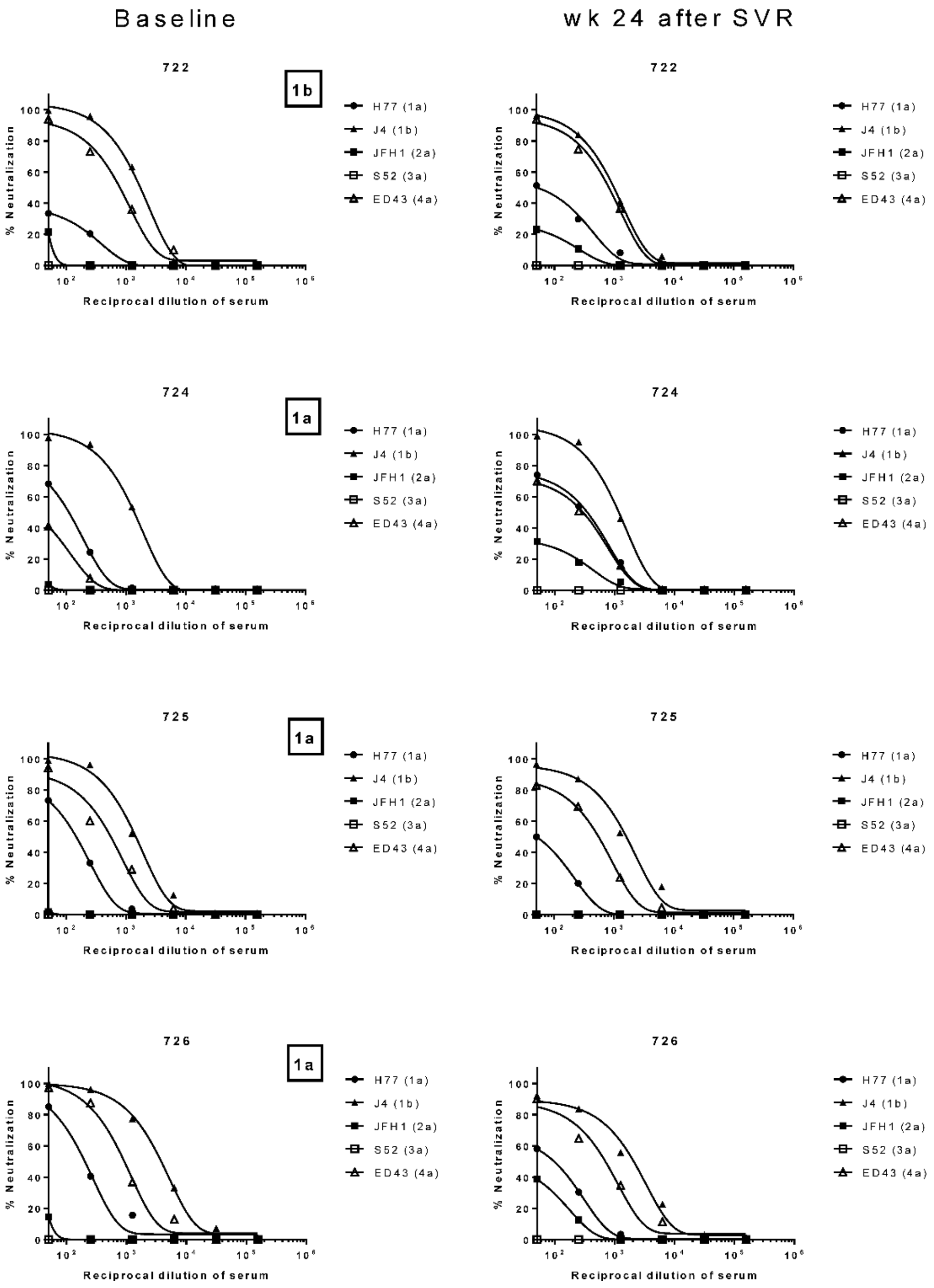
Baseline
wk 24 after SVR
722
1b
724
1a
725
1a
726
1a
H77 (1a)
J4 (1b)
JFH1 (2a)
S52 (3a)
ED43 (4a)
% Neutralization
Reciprocal dilution of serum

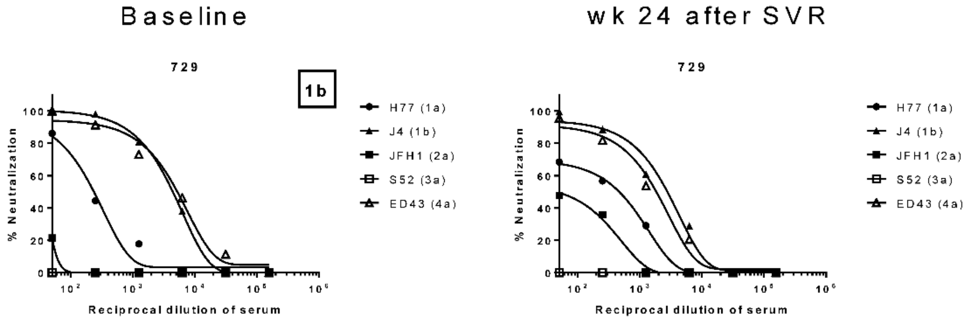
Baseline
wk 24 after SVR
729
1b
% Neutralization
Reciprocal dilution of serum
H77 (1a)
J4 (1b)
JFH1 (2a)
S52 (3a)
ED43 (4a)

Supplementary Figure 2. Phylogenetic analyses of E1E2 nucleotide sequences from A) chimeric HCV and HCV GT1a infected patients and B) HCV GT1 and HCV GT1a infected patients. The percentage of replicate trees in which the associated virus clustered together in the bootstrap test (1000 replicates) are shown next to the branches.

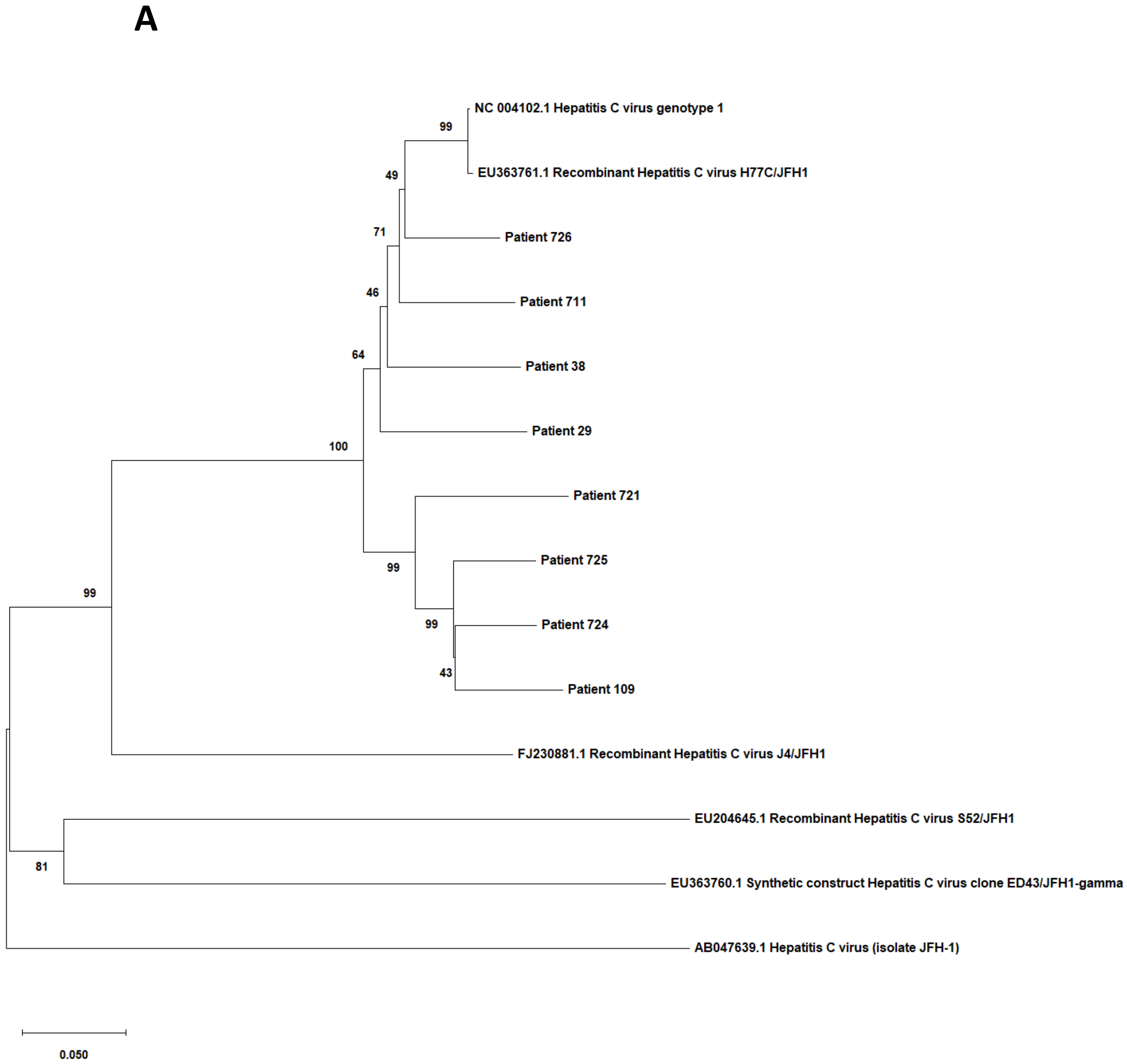

B

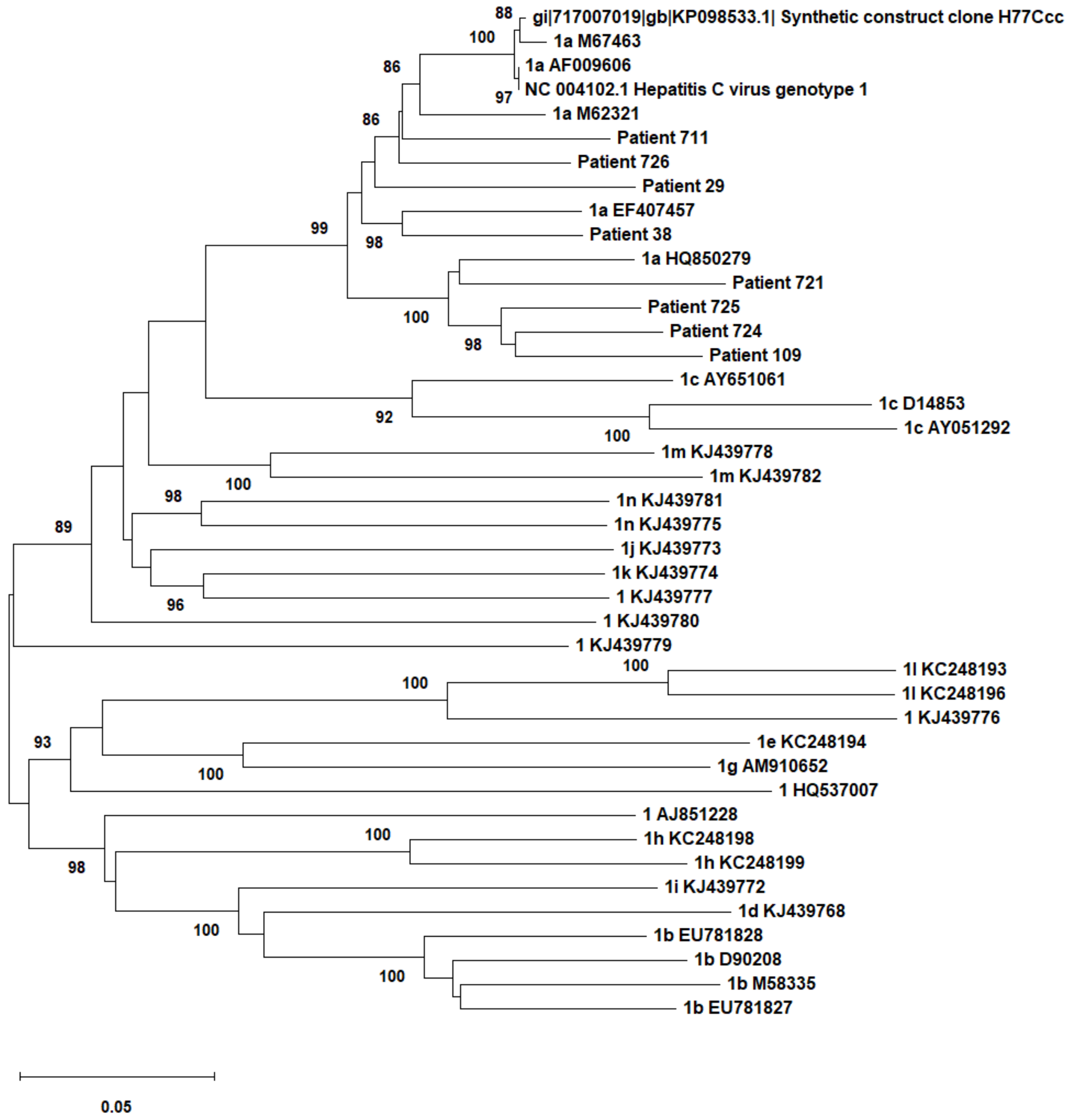
gi|717007019|gb|KP098533.1| Synthetic construct clone H77Ccc
1a M67463
1a AF009606
NC 004102.1 Hepatitis C virus genotype 1
1a M62321
Patient 711
Patient 726
Patient 29
1a EF407457
Patient 38
1a HQ850279
Patient 721
Patient 725
Patient 724
Patient 109
1c AY651061
1c D14853
1c AY051292
1m KJ439778
1m KJ439782
1n KJ439781
1n KJ439775
1j KJ439773
1k KJ439774
1 KJ439777
1 KJ439780
1 KJ439779
1l KC248193
1l KC248196
1 KJ439776
1e KC248194
1g AM910652
1 HQ537007
1 AJ851228
1h KC248198
1h KC248199
1i KJ439772
1d KJ439768
1b EU781828
1b D90208
1b M58335
1b EU781827
88
100
97
86
86
99
98
100
98
92
100
100
98
89
96
100
100
93
100
100
98
100
100
0.05

Supplementary Figure 3. Antibody titers against the E2 glycoprotein of H77 (HCV-GT1a). Serial dilutions (dilution factor 2, initial dilution 1:50) of plasma samples were tested against purified E2 glycoprotein by an enzyme-linked immunosorbent assay (ELISA). Patient numbers are shown. Curves represent one phase exponential decay least squares fit.

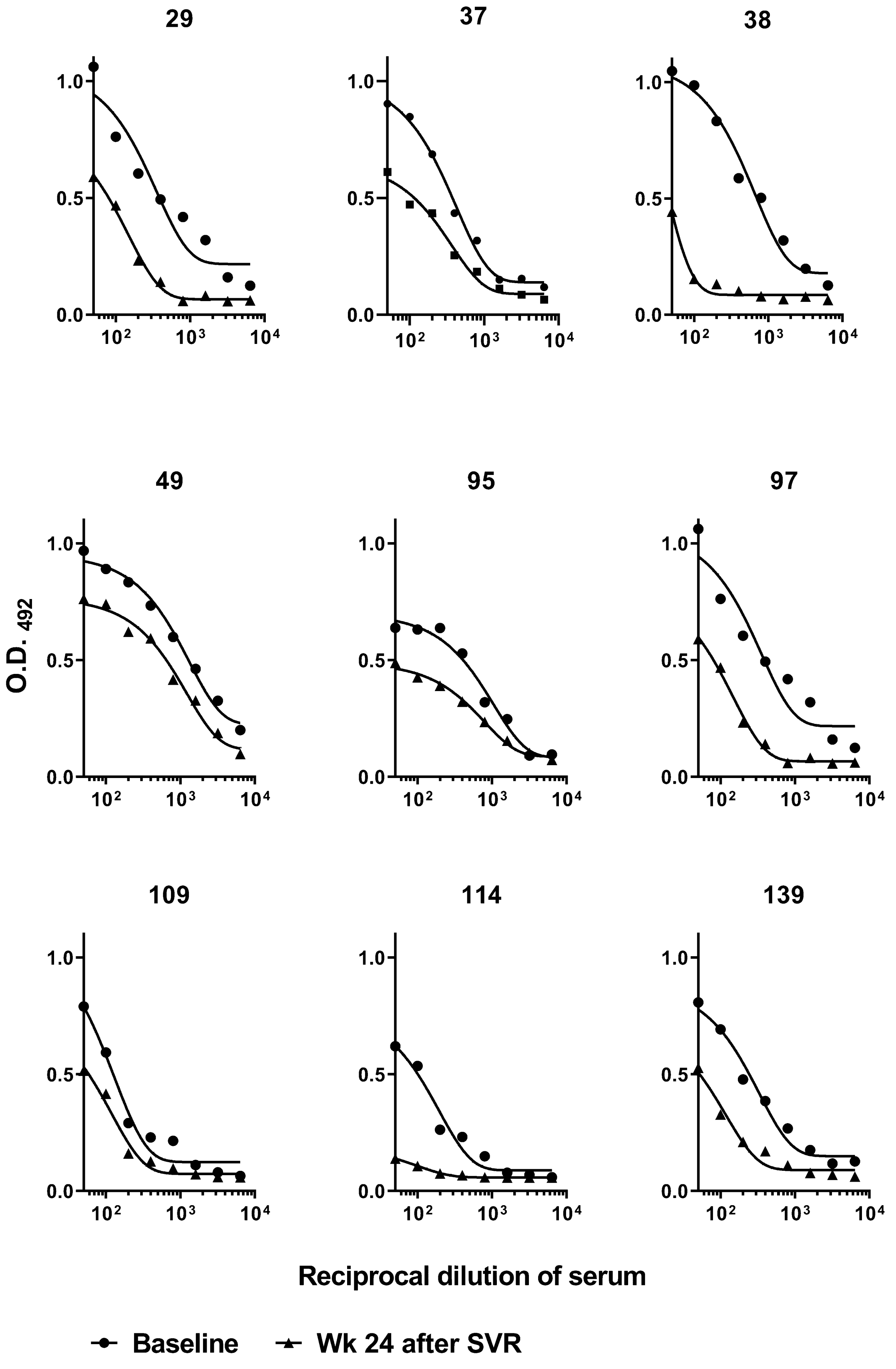

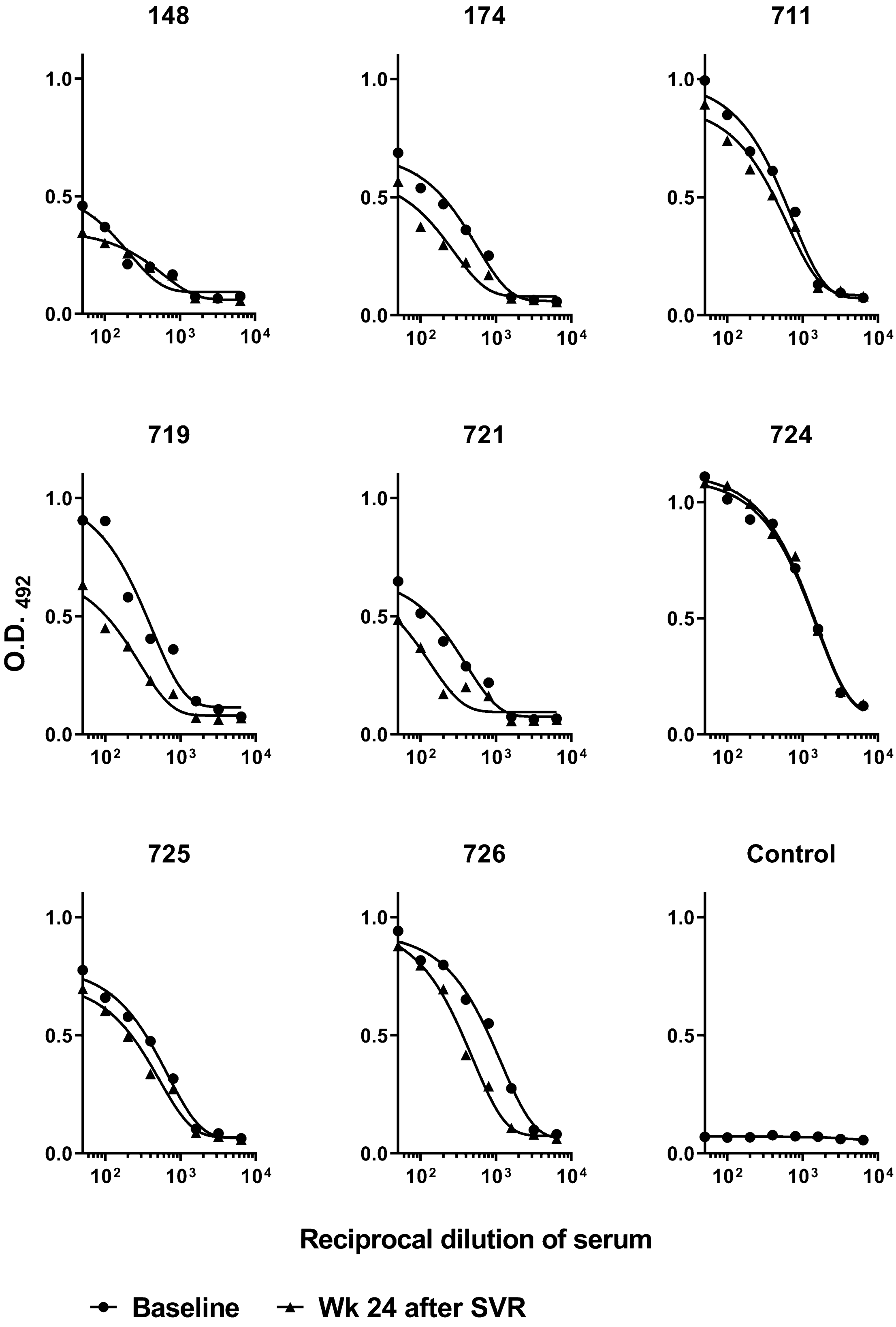
148
174
711
719
721
724
725
726
Control
O.D. 492
1.0
0.5
0.0
10²
10³
10⁴
Reciprocal dilution of serum
Baseline
Wk 24 after SVR

Supplementary Table 1. AUC values against recombinant viruses at baseline and at week 24 after SVR in HIV/HCV-coinfected study patients.

		H77 (1a)		J4 (1b)		JFH1 (2a)		S52 (3a)		ED43 (4a)	
**Patient #**	**Genotype**	**Baseline**	**Week 24 after SVR**	**Baseline**	**Week 24 after SVR**	**Baseline**	**Week 24 after SVR**	**Baseline**	**Week 24 after SVR**	**Baseline**	**Week 24 after SVR**
29	1a / 2	34970	24100	597330	138000	75320	2150	16270	0	199300	81450
37	1a	723640	230	4120000	281140	413950	0	14240	0	2239840	41600
38	1a / 4	71940	51970	866620	722600	12800	0	0	0	371440	387130
48	3	167550	67800	828920	197920	29270	1600	0	0	279510	78240
49	1a	38360	17060	410820	486060	190	0	0	0	192550	189080
95	1a / 1b	18240	9140	1956040	190500	115170	0	0	0	936520	61770
96	3	21110	48710	142310	38640	7140	1280	0	0	88230	78040
97	1a	107130	11060	176740	25670	75960	1730	290	0	85210	1110
103	1b	44320	62200	583600	127460	176730	2340	0	0	66670	62650
109	1a	30470	61420	618680	259660	44410	72060	0	0	108900	84940
114	1a	491440	77440	4752450	448990	168810	1290	11400	0	672060	420000
115	3	25600	93080	226150	212760	370	29940	0	0	8660	85420
133	3	92140	14800	396760	45760	37920	1180	1000	0	83620	13160
139	1a	19030	54740	344900	164950	1440	14650	0	0	370770	127930
148	1a	15650	47620	300770	272040	2160	8790	0	0	311200	164320
174	4 / 1a	25720	76390	818800	426700	69470	3550	3000	0	423500	110110
213	3	302530	108220	1227940	226780	600440	9700	7640	0	1927420	195500
216	3 / 4	70200	19390	1259620	154260	57920	0	0	0	199600	79670
432	3	0	0	0	0	0	0	0	0	0	0
515	3	12190	9760	199200	93740	1380	380	0	0	117130	117290
711	1a	188730	76200	2680110	922120	214880	18030	0	0	1551220	233750
718	3	3750	18880	301860	439680	21600	23560	0	0	148950	481490
719	1a	130780	4050	1825180	104530	100740	0	0	0	449400	292230
721	1a	18770	6260	18620	2630	4720	50	0	0	26580	1
722	1b	0	330	45240	2740	1840	0	0	0	164260	1
724	1a	109270	16820	1690600	474000	73160	1	0	0	657580	324500
725	1a	79660	33960	1337920	783350	1450	11360	0	0	369420	322990
726	1a	88650	128160	881750	681050	2150	26370	0	0	1826080	529100
729	1b	90390	22060	564350	65180	52970	1	0	0	490260	233940

Abbreviations: AUC, Area under the neutralization curve; SVR, sustained virological response.

Supplementary Table 2. ID50 values against recombinant viruses at baseline and at week 24 after SVR in HIV/HCV-coinfected study patients.

		H77 (1a)		J4 (1b)		JFH1 (2a)		S52 (3a)		ED43 (4a)	
Patient #	Genotype	Baseline	Week 24 after SVR	Baseline	Week 24 after SVR	Baseline	Week 24 after SVR	Baseline	Week 24 after SVR	Baseline	Week 24 after SVR
29	1a / 2	187.8	77.1	2699.9	688.1	0	0	0	0	1118.6	190.9
37	1a	2183.0	0	19760.3	413.1	1656.8	0	0	0	8080.0	1
38	1a / 4	224.4	0	3453.7	3060.6	0	0	0	0	1162.6	1756.5
48	3	671.7	167.8	4029.7	1093.6	124.9	0	0	0	1139.7	384.5
49	1a	147.3	50.0	1463.6	1600.2	0	0	0	0	558.8	549.2
95	1a / 1b	54.7	0	5840.7	1022.7	399.9	0	0	0	4831.2	263.6
96	3	106.2	0	707.6	242.9	0	0	0	0	511.5	303.9
97	1a	401.1	52.9	928.2	146.4	207.8	0	0	0	482.5	0
103	1b	195.9	115.7	2481.9	666.0	486.1	0	0	0	352.5	144.7
109	1a	0	246.1	2860.1	958.0	0	0	0	0	530.8	368.1
114	1a	2169.8	327.2	21285.0	1908.0	848.4	0	0	0	2841.3	1257.0
115	3	110.1	346.4	1365.8	1168.1	0	0	0	0	26.5	294.4
133	3	535.9	0	2056.2	152.8	63.3	0	0	0	443.1	0
139	1a	0	0	1026.7	660.2	0	0	0	0	1087.2	572.5
148	1a	0	50.6	1732.9	987.5	0	0	0	0	789.1	813.7
174	4 / 1a	152.2	339.5	3666.0	1327.6	342.0	0	0	0	1096.5	488.5
213	3	890.4	454.9	5399.1	1368.4	2296.9	0	0	0	7486.5	1108.1
216	3 / 4	330.0	0	5025.9	771.7	120.2	0	0	0	1111.2	360.8
432	3	0	0	0	0	0	0	0	0	0	0
515	3	0	0	1114.5	460.2	0	0	0	0	487.6	435.9
711	1a	697.1	285.8	12429.1	4755.3	750.9	0	0	0	6049.9	1129.8
718	3	0	73.1	1686.4	1507.7	57.0	0	0	0	752.7	1437.1
719	1a	596.9	0	7168.9	529.2	96.9	0	0	0	1507.8	936.8
721	1a	0	0	0	0	0	0	0	0	56.8	0
722	1b	0	0	277.7	0	0	0	0	0	432.9	0
724	1a	413.8	0	5299.1	1249.9	186.2	0	0	0	2981.5	901.2
725	1a	200.9	96.9	3784.1	2070.1	0	0	0	0	916.0	696.5
726	1a	236.4	464.6	4420.2	2772.1	0	0	0	0	5219.7	1839.0
729	1b	403.8	0	2435.3	354.3	250.2	0	0	0	1110.6	701.4

Abbreviations: ID50, 50% inhibitory dose; SVR, sustained virological response.

Supplementary Table 3. Relationship between the CD4/CD8 ratio and the HCV-nAb titers against recombinant viruses in HIV/HCV-coinfected study patients.

		AUC		ID50	
	**HCV genotypes**	**r**	**p-value ***	**r**	**p-value ***
**At baseline**	**H77 (1a)**	0.454	**0.013**	0.450	**0.014**
	**J4 (1b)**	0.248	0.194	0.246	0.198
	**JFH1 (2a)**	0.192	0.318	0.142	0.462
	**S52 (3a)**	0.042	0.830	-	-
	**ED43 (4a)**	0.002	0.992	0.125	0.519
**At week 24 after SVR**	**H77 (1a)**	-0.115	0.553	-0.056	0.772
	**J4 (1b)**	0.041	0.833	-0.082	0.671
	**JFH1 (2a)**	-0.133	0.492	-	-
	**S52 (3a)**	-	-	-	-
	**ED43 (4a)**	-0.158	0.414	-0.209	0.276

Abbreviations: HCV-nAbs, HCV neutralizing antibodies; r, Spearman correlation coefficient; AUC, area under the curve; ID50, 50% inhibitory dose; SVR, sustained virological response.

Statistics: p-values were calculated by the Spearman correlation coefficient.
